# Core-Shell Structured HMX@Polydopamine Energetic Microspheres: Synergistically Enhanced Mechanical, Thermal, and Safety Performances

**DOI:** 10.3390/polym11030568

**Published:** 2019-03-26

**Authors:** Congmei Lin, Feiyan Gong, Zhijian Yang, Xu Zhao, Yubin Li, Chengcheng Zeng, Jiang Li, Shaoyun Guo

**Affiliations:** 1 Institute of Chemical Material, China Academy of Engineering Physics, Mianyang 621900, China; lincmei2009@caep.cn (C.L.); xuzhao@caep.cn (X.Z.); liyubin030102@caep.cn (Y.L.); zengcc1314@caep.cn (C.Z.); 2 The State Key Laboratory of Polymer Materials Engineering, Polymer Research Institute of Sichuan University, Chengdu 610065, China; li_jiang@scu.edu.cn (J.L.); nic7702@scu.edu.cn (S.G.)

**Keywords:** bio-inspired interfaces, mechanical properties, thermal stability, sensitivity

## Abstract

The solid–solid phase transition, poor mechanical properties, and high sensitivity has impeded further practical applications of 1,3,5,7-tetranitro-1,3,5,7-tetrazocane (HMX) based polymer bonded explosives (PBXs). To address these issues together, a facile and effective route was employed to achieve a coating of polydopamine (PDA) on the surface of explosive crystals via in situ polymerization of dopamine. Additionally, PBXs based on HMX@PDA microcapsules were prepared with a fluoropolymer as polymer binder. Improved storage modulus, static mechanical strength and toughness, and creep resistance has been achieved in as-prepared PDA modified PBXs. The β-δ phase transition temperature of as-obtained PBXs based on conventional HMX (C-HMX)@PDA was improved by 16.3 °C. The friction sensitivity of the C-HMX based PBXs showed a dramatic drop after the PDA coating. A favorable balance proposed in this paper among thermal stability, mechanical properties, and sensitivity was achieved for C-HMX based PBXs with the incorporation of PDA.

## 1. Introduction

Energetic materials (EMs) contain high chemical energy, in which organic small molecular crystals act as functional materials. For decades, they have attracted increasing interest for their significant applications in both military and civil fields. Among them, 1,3,5,7-tetranitro-1,3,5,7-tetrazocane (HMX) and HMX-based formulations have been extensively studied, due to the high detonation energy and high melting point [1,2]. However, the high sensitivity towards external mechanical stimuli, phase transition under thermal insult, and poor mechanical properties induced by weak adhesive properties with polymer binder are main shortcomings which limit its applications.

Several strategies have been established to desensitize HMX, including preparing HMX with higher crystal quality by recrystallization [3], designing energetic cocrystals [4], synthesizing HMX@insensitive explosive core-shell microparticles [5,6], and coating the HMX crystal with polymers via in situ polymerization [7]. However, the energetic cocrystals and core-shell microparticles which combine the HMX with large amounts of insensitive explosive reduce the energy output. Due to the difficulty in controlling the mass transfer, the polymer binder cannot be completely coated onto explosive crystals at the large-scale production by the in situ polymerization.

Another limitation for the application of HMX energetic crystal is that it undergoes a solid–solid β-δ phase transition under thermal shock, which is harmful for the long-term storage and transportation of materials. Solid–solid phase transition causes expansion [8] and extensive microstructural damage [9], such as mesoscale evolution of voids and porosity, which tend to act as hot spots under external impact or shock, making the heated HMX based explosives more sensitive [10,11]. So far, coating approaches have been mainly developed to tune the phase transition temperature of the explosives [12]. Additives of TATB and olefin in high concentration can form compact coatings on the HMX crystals to delay the nucleations of δ-HMX and build up a heat conduction obstacle, leading to a higher temperature required for the β-δ phase transition [13]. The phase transition of HMX explosives can be greatly improved after core-shell coating of melamine-formaldehyde resin, with the appreciable increment of more than 16 °C [7]. However, the high concentration of shell materials is necessary to completely coat explosive crystals. In contrast, fewer additives lead to larger free surface area of HMX, which accelerates the phase transition [13]. Furthermore, solid–solid phase transition of explosive crystals depends on the chemical interaction between explosives and binders, which may promote or delay the phase transition [14].

Polymer bonded explosives (PBXs) are typical polymer-based energetic composites with a high loading of solid explosive crystals. The mechanical properties of PBX composites are determined in part by the chemical structure and bonding at the interface between constituents in the microstructure [15]. Mechanical failure paths tend to primarily run around the interface between crystals and the binder matrix and avoid regions of fine filler and binder [16]. This can lead to materials with abundant cracks, which is harmful for the safety and reliability of the explosive. Considerable efforts have been undertaken to increase the interfacial interaction and mechanical properties of HMX and HMX-based composites. A common and convenient method is the addition of neutral polymer bonding agent (NPBA) to achieve interfacial reinforcement [17]. In situ polymerization of hydroxyethyl acrylate-acrylate-acrylonitrile copolymer and isophorone diisocyanate is also applied to the coating on HMX surfaces [18]. It is shown that forming a cross-linked polymeric coating on HMX plays a role for interfacial reinforcement between HMX fillers and polymeric binders, resulting in a 21% increase of the tensile strength. Another technique is the fabrication of core-shell microparticles, such as HMX@nano-TATB composites [19]. It significantly changes the surface morphology of HMX and the interface adhesion state between particles and polymer binder, resulting in the enhancement of mechanical properties. Among them, it is still a big challenge to precisely control the surface structure and morphology for the application of NPBA and in situ polymerization. Besides, the production scale of HMX@nano-TATB core-shell particles is difficult to amplify for engineering applications, due to the extraordinary low solubility of TATB in common solvents.

Up until now, mussel-inspired coating with the in-situ polymerization of dopamine has raised increasing interest and has been frequently used to coat various substrates, such as glass, metal, polymer, and nanocarbon materials [20,21,22,23,24,25,26,27,28]. Recently, there have been some reports on the application of dopamine chemistry to energetic crystal processing, such as HMX, hexanitrohexaazaisowurtzitane (CL-20), and 1,3,5-triamino-2,4,6-trinitrobenzene (TATB) [29,30,31]. It has been found that in situ polymerization of dopamine can provide a facile and versatile method for modifying the surfaces of energetic crystals. However, little work has been done to explore the application of well-coated energetic crystals in PBXs and its effects on the comprehensive performance.

The balance among the explosive performances, including the sensitivity, thermal stability, and mechanical properties of the energetic material, is fraught with challenges. Such three performances of PBX often constrain each other, and it is very difficult to synergistically improve. Therefore, novel techniques to acquire better balance and super comprehensive performance should be explored. The strong chemical adhesion to form the robust and compact core-shell structure and high rigidity of polydopamine (PDA) enables it to provide a great potential to efficiently reduce the sensitivity and improve the thermal stability and mechanical properties of energetic materials without a sacrifice of detonation power. In this work, we demonstrate a facile in situ polymerization approach to synthesize core-shell microparticles with a high-energy HMX core and a PDA shell. Then, the polymer binder was added to further coat HMX@PDA microspheres and prepare PBXs. The crystal quality and particle size of HMX crystals are varied to study their effects on the thermal and mechanical behavior of PBXs. Additionally, the amount of PDA in core-shell microparticles is varied to investigate the dependence of the comprehensive properties on the degree of coating.

## 2. Experimental Section

### 2.1. Materials

Three kinds of HMX were used, including a conventional one of industrial grade (C-HMX) and two recrystallized samples with reduced sensitivity (RS-HMX). The C-HMX was purchased (Baiyin Chemical Industry Co., Ltd., Baiyin, China) and used without further purification. RS-HMX with fine and large grains were marked as FRS-HMX and LRS-HMX, respectively. Dopamine and (hydroxymethyl)aminomethane (Tris) were obtained from Sigma-Aldrich (St. Louis, MI, USA) and used as received. Ultrapure water with a resistivity of 18.2 MΩ·cm was prepared with a Milli-Q apparatus (Millipore, Billerica, MA, USA). A vinylidene fluoride (VDF) and chlorotrifluoroethylene (CTFE) copolymer provided by Zhonghao Chenguang Chemical Industry Co., Ltd. (Zigong, China) was used as polymer binders.

### 2.2. Sample Preparations

**Figure 1:**
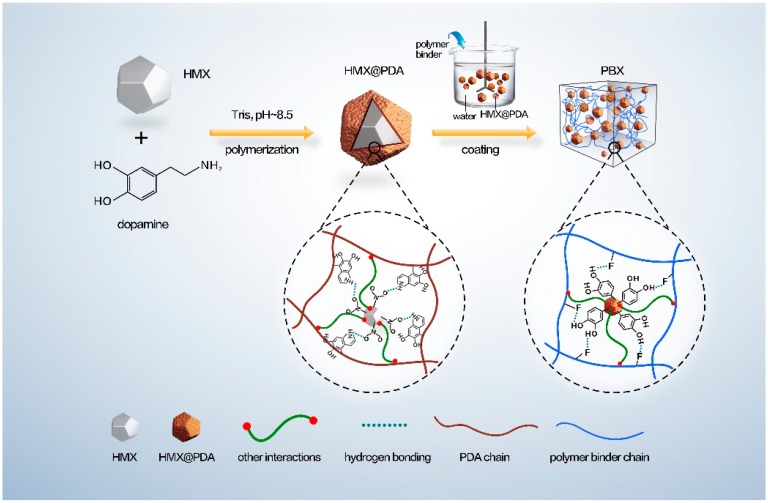
Proposed schematic preparation procedure and enhancement mechanism for polydopamine (PDA) modified polymer bonded explosives (PBXs).

The preparation of core-shell structured HMX@PDA and PBX composites are illustrated in Figure 1. The PDA-coated HMX were synthesized as follows: Tris solution (10 mM) was prepared and adjusted to pH of 8.5 by HCl solution. 100 g HMX crystals were added to 3000 mL Tris solution and dispersed under high-intensity ultrasonic irradiation for 5 min. Then, 6 g dopamine was added to the suspension whilst stirring at room temperature. After filtering and washing with a large quantity of ultrapure water several times to remove excessive PDA in the solution, the PDA-coated HMX particles were obtained by drying at 60 °C in a vacuum oven. In this paper, samples were denoted as *x*HMX@PDA-*y*h, where *x* represented the HMX particle type and *y* represented the PDA coating time. For example, C-HMX@PDA-3h represented the matrix was C-HMX and the corresponding PDA coating time was 3 h.

The molding powders of various PBX formulations were prepared by water suspension method. For distinguishing purposes, PBX based on C-HMX, FRS-HMX, and LRS-HMX were labeled as PBX-C-yh, PBX-F-yh, and PBX-L-yh, respectively, where *y* represented PDA coating time. For instance, PBX-C-3h represented PBX based on the C-HMX with the corresponding PDA coating time of 3 h. The obtained molding powders were then dried in a vacuum oven at 60 °C for 48 h. To test the mechanical and creep properties of the PBXs, the molding powders were pressed in a mold and transformed into explosive pellets with a given geometrical shape.

### 2.3. Material Characterizations

The coating content of the PDA shell was quantitatively analyzed by a high-performance liquid chromatography (HPLC, Agilent, Santa Clara, CA, USA). The morphologies and structures of various HMX@PDA samples were characterized by matching refractive index (OMMRI), scanning electron microscopy (SEM, Zeiss, Oberkochen, Germany), X-ray photoelectron energy spectrum (XPS, Thermo Fisher Scientific, Waltham, MA, USA), laser particle size analyzer (LPSA, Beckman Coulter, Brea, CA, USA), Fourier-transform infrared spectra (FT-IR, Thermo Fisher Scientific, Waltham, MA, USA), Raman spectra (Renishaw, Gloucestershire, UK), and X-ray diffraction (XRD, Bruker, Karlsruhe, Germany). The thermal properties of the HMX and corresponding PBXs were analyzed by using a differential scanning calorimeter (DSC, Mettler, Zurich, Swiss). The mechanical reinforcement effect of PDA on various HMX and PBX composites was investigated by compressive stiffness tests (CST), dynamic mechanical analysis (DMA), creep, Brazilian, and compression tests. The mechanical sensitivity measurements were conducted according to GJB-772A-97 standard method 601.2 and 602.1 [32]. Detailed characterization methods and processes are shown in Electronic Supplementary Information (ESI).

## 3. Results and Discussion

### 3.1. Morphological and Structural Features of Core-Shell HMX@PDA Particles

The morphologies of three HMX raw crystals are exhibited in Figure S1 in ESI. RS-HMX crystals possessed a more regular diamond-like shape, less internal defects, and a narrower particle size distribution than that of C-HMX. The amount of PDA for core-shell HMX@PDA can be tuned by simply varying the coating time (Table S1 in ESI). With increased coating time, the white HMX crystals gradually changed from gray to thick brown with increasing PDA coating amount (Figure S2 in ESI). The average particle size of C-HMX, FRS-HMX, and LRS-HMX was 47.0, 44.8, and 149.1 μm, respectively. Compared with the raw materials, the average particle size for the FRS-HMX@PDA became smaller, suggesting the better dispersion of the particle powders due to the PDA coating.

**Figure 2:**
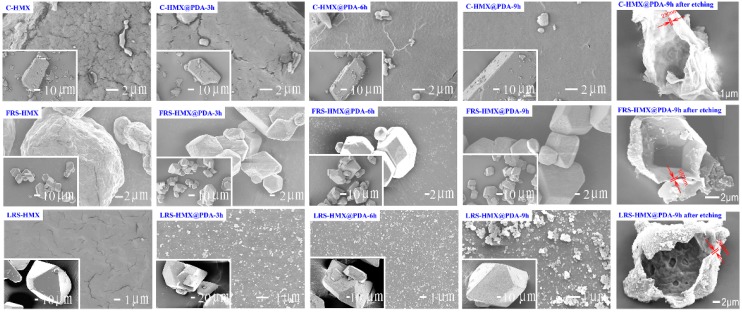
SEM micrographs of the structure morphologies for different HMX and PDA coated specimens.

The surface morphologies of the raw HMX materials and PDA coated HMX crystals were investigated in detail using SEM measurements, as shown in Figure 2. The surface morphologies of the C-HMX and FRS-HMX crystals exhibited ignorable change after PDA modification. It may be attributable to the difference in surface features and specific surface area. However, the color change of C-HMX and FRS-HMX crystals with the polymerization time was similar to that of LRS-HMX@PDA composites (Figure S2 in ESI). The white HMX crystals gradually changed from pale brown to a deep brown with the increase of PDA contents. Furthermore, the resistance ability to electron beams was also gradually increased, as examined by the C-HMX crystals. Neat HMX was sensitive to electron beams and a lot of cracks were observed on the crystal surface. With a uniform and compact PDA coating shell, the amount of cracks on the crystal surface gradually relieved, indicating that the PDA coating could enhance the high power electron beam bombardment resistance of HMX. The influences of electron beam time on the evolution of surface morphology for C-HMX@PDA-9h composites were also investigated and depicted in Figure S3 in ESI. Undistinguishable cracks could be observed after exposing C-HMX@PDA-9h under electron beam for 3 min, which could be possibly attributed to the protection of PDA.

In addition, the surface of LRS-HMX crystals was smooth, while LRS-HMX@PDA was observed as a relatively rough surface made up of compact and continuous PDA shell. It can be seen from Figure 2 that more PDA particles and a thicker PDA coating layer formed on the LRS-HMX surface with the processing time increasing from 3 to 6 h, building up a uniform and compact shell. Moreover, PDA agglomeration could be obviously observed on the LRS-HMX surface when the reaction time increased to 9 h. 

A core-etching technique was introduced, using acetone as the etching solvent. SEM characterization of the indiscerptible PDA shells was carried out and the results are also shown in Figure 2, exhibiting a hollow PDA shell structure of HMX@PDA after the etching treatment. The PDA shell maintained the shape of the HMX crystal, with the inner surface reflecting the roughness of interface between PDA and HMX. The shape of particles of C-HMX samples was irregular with a rough surface, while a relatively smooth surface for RS-HMX can be obtained. Furthermore, for core-shell samples, the thickness of PDA shell was at nano scale, depending on the HMX matrix.

**Figure 3:**
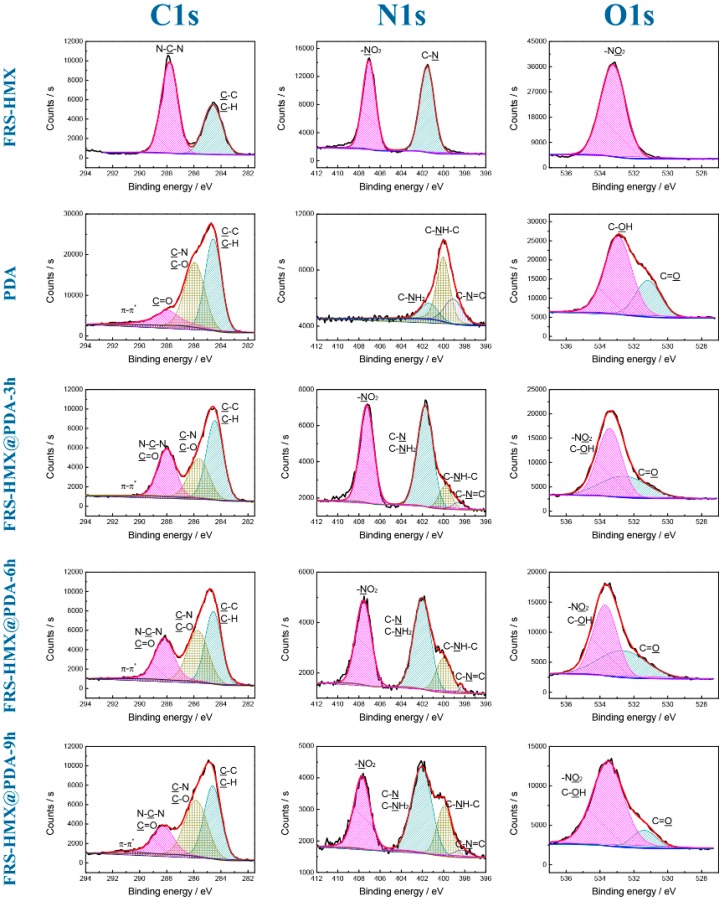
XPS of the uncoated FRS-HMX crystals, PDA, and FRS-HMX@PDA microparticles.

Element states on the surface of energetic microcapsules could be conducted by XPS analyses [33]. The C 1s, O 1s, and N 1s spectra for FRS-HMX, PDA, and various FRS-HMX@PDA samples are shown in Figure 3. The characteristic peaks of C 1s, O 1s, and N 1s spectra for HMX and PDA were in accordance with the results of the previous reports [5,34]. The corresponding functional groups of PDA coated composites are marked in Figure 3.

According to quantitative analysis, the surface element composition of FRS-HMX, PDA, and FRS-HMX@PDA composites are listed in Table S2 in ESI. The coating efficiency of PDA can be also estimated from XPS results by the change of N/C ratio. The N/C ratio on the surface of FRS-HMX@PDA composites was gradually close to that of PDA, indicating the successful surface modification by PDA. With the increase in polymerization time from 3 to 9 h, the decrease in N/C ratio from 0.485 to 0.287 indicated the gradual deposition of PDA content on the surface of microcapsules. XPS atomic concentration of functional groups in all atoms of HMX, PDA, and HMX@PDA are summarized in Table S3 in ESI. The concentration ratios of C–NH–C group (in PDA)/NO_2_ group (in FRS-HMX) from N 1s spectrum were 0.21, 0.40, and 0.58 for FRS-HMX@PDA-3h, FRS-HMX@PDA-6h, FRS-HMX@PDA-9h composites, respectively. The results indicated a successful coating of PDA on the FRS-HMX step by step. Hence, the perfect core-shell structure of HMX@PDA microcapsules was further confirmed by XPS.

The crystal structures were further investigated by Raman spectrum, X-ray diffractometry (XRD) patterns, and Fourier-transform infrared (FT-IR) spectra. After surface modification by dopamine solution, a new absorption signal from Raman spectrum in Figure S4a appeared at 1580–1700 cm^−1^, which can be attributable to the typical feature of G bands in PDA [35]. In Figure S4b, all diffraction peaks of HMX and HMX/PDA can be indexed to β-HMX (JCPDS card No. 42-1768), indicating the polymorph nature of the crystals did not change. FT-IR results in Figure S4c indicated that the PDA coated composites displayed a combination of the characteristic peaks of the functional groups in HMX [36] and PDA [37]. In addition, the values of the initial secant modulus (ISM) calculated according to Ref. [38] were dependent on the PDA content (Figure S4d and Table S1 in ESI). Due to the enhancement of the density of cross-linked network, the ISM of these core-shell HMX@PDA composites increased with increasing the content of PDA.

### 3.2. Detonation Properties of PBXs Based on HMX@Polydopamine

According to the Urizar equation, the detonation velocity of composites explosive (*D*) could be calculated by:

(1) $$w_{i}=\frac{V_{i}}{\sum V_{i}}$$

(2) $$D={\sum w}_{i}D_{i}$$

where *V_i_* denotes the volume of component *i*, *w_i_* is the volume fraction of component *i*, and *D_i_* is the characteristic detonation velocity of component *i*, respectively. The characteristic detonation velocity for HMX and insensitive materials (PDA and fluoropolymer) are about 9150 and 5400 m/s, respectively. In order to maintain detonation performance unchanged, the mass fraction of HMX in the formulation remained a certain value of 95%. The calculated theoretical detonation velocity is listed in Table S4. It can be found that the difference of detonation velocity of all the formulations was less than 0.32%.

### 3.3. Mechanical Properties of PBXs Based on HMX@Polydopamine

Figure S5 reflects the dependence of the dynamic mechanical properties, including storage modulus (*E’*) and loss factor (tan δ) on the temperature for PDA modified PBXs. With the incorporation of PDA, the value of storage modulus was improved due to the enhanced interface. An inflexion, corresponding to the T_g_ of corresponding fluoropolymer binders [39], could be observed in the loss factor curves between 45–75 °C for the PBXs.

**Figure 4:**
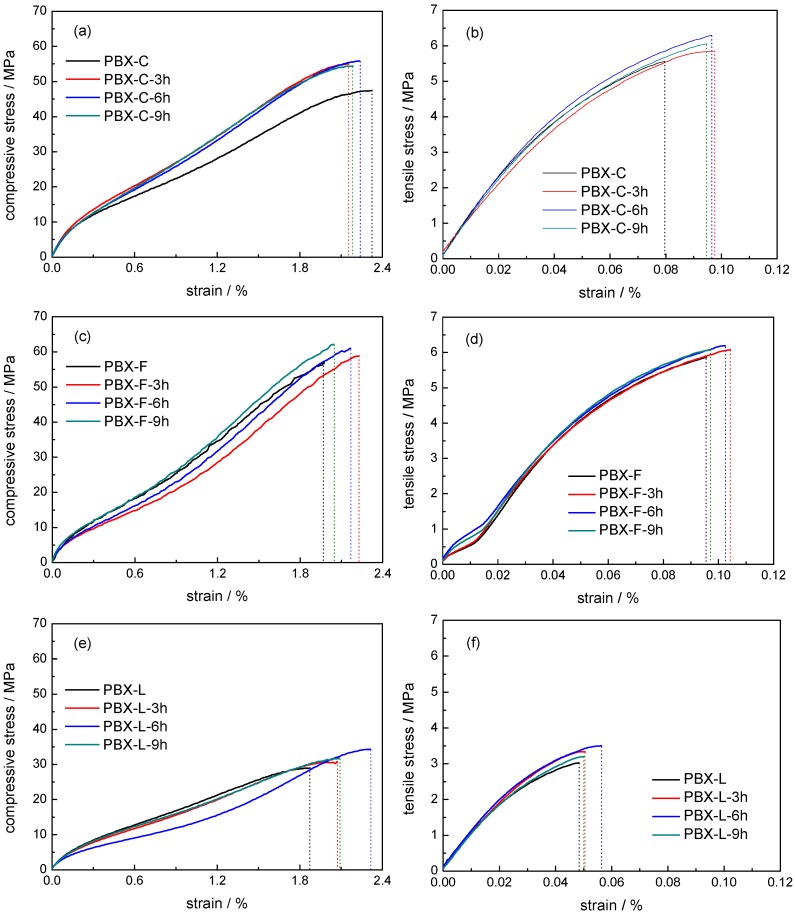
The typical mechanical response at room temperature for PDA modified PBXs: compressive test (**a**,**c**,**e**); Brazilian test (**b**,**d**,**f**).

Static compressive and Brazilian tests were also conducted to analyze the effect of PDA modification on the mechanical properties of PBXs. Representative mechanical characteristics were summarized in Figure 4 and Table S4 in ESI, indicating an obvious enhanced mechanical property after PDA modification. Generally, the compressive and tensile strength and elongation at break were higher than the corresponding samples without PDA coating, suggesting a reinforcing and toughening role of PDA in PBX. The compressive fracture energy (*W_c_*) and the tensile fracture energy (*W_t_*), obtained by the integration of the stress–strain curves, could be used to characterize the toughness of explosive materials. PBXs with a PDA polymerization time of 6 h presented optimal mechanical properties. PBX-C-6h, PBX-F-6h, and PBX-L-6h composites showed 40.1%, 17.4%, and 38.4% improvement in *W_t_* compared to the corresponding pristine PBX-C, PBX-F, and PBX-L samples, respectively.

**Figure 5:**
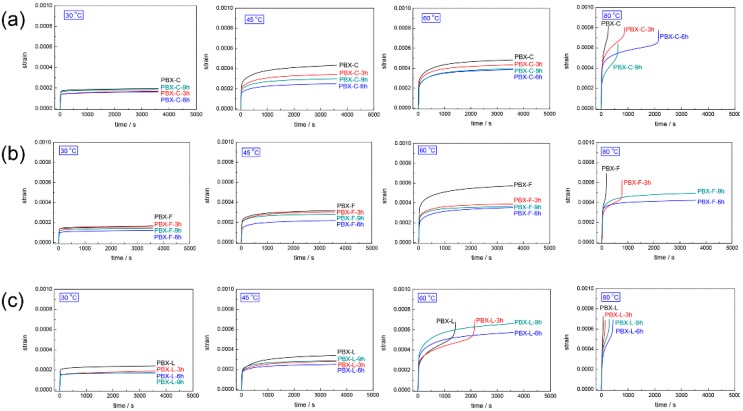
Time-dependent creep strain of PBXs at different temperatures under 4 MPa: (**a**) PDA modified PBX-C, (**b**) PDA modified PBX-F, (**c**) PDA modified PBX-L.

Consequently, creep analysis was applied to analyze the effects of PDA content on the non-linear viscoelastic properties for PBXs. As shown in Figure 5, the introduction of PDA significantly decreased the creep strain at low temperatures (30 and 45 °C) and prolonged the creep failure time at high temperatures (60 and 80 °C), illustrating the reinforcing effect of PDA on the creep resistance of the materials. Obviously, PBXs with PDA polymerization time of 6 h exhibited an excellent improvement in creep resistance, which agreed with the above dynamic and static mechanical results. It can be associated with the significant improvement interfacial interaction between HMX crystals and polymer binders, achieved by the incorporation of PDA, and thus restriction of the mobility of polymer chains.

### 3.4. Mechanism for the Enhancement of Mechanical Properties

To summarize the synthetic procedure of PDA shell and enhancement mechanism in PBXs, a schematic mechanism was proposed in Figure 1. The gradual in situ polymerization of dopamine was coated or deposited on the HMX crystal surface. Sufficient noncovalent interactions, including π–π stacking, charge transfer, and hydrogen bonding between the N–O groups in the HMX molecules and the catechol groups in the PDA chains, were carried out between the PDA and HMX crystals [32]. Subsequently, the compact and uniform PDA shell was formed to give core-shell energetic microcapsules.

As the polymer binder was added, the PDA layer acted as an interfacial platform to construct a bridge between HMX and fluoropolymer. Sufficient interaction, including hydrogen bonding interactions and other interactions, would be carried out between the PDA molecules and HMX explosives. The supramolecular interactions, such as hydrogen bonding etc., could contribute to and be important interaction mechanisms between PDA and fluoropolymers. The hydrogen bonds with the OH groups as proton donors and F groups in the fluoropolymer chains as proton acceptors constructed a physical cross-linking network [40]. As a result, PDA formed multiple interfacial interactions with HMX crystal and fluoropolymer binders to modify mechanical behavior.

### 3.5. Thermal Properties of PBXs Based on HMX@Polydopamine

**Figure 6:**
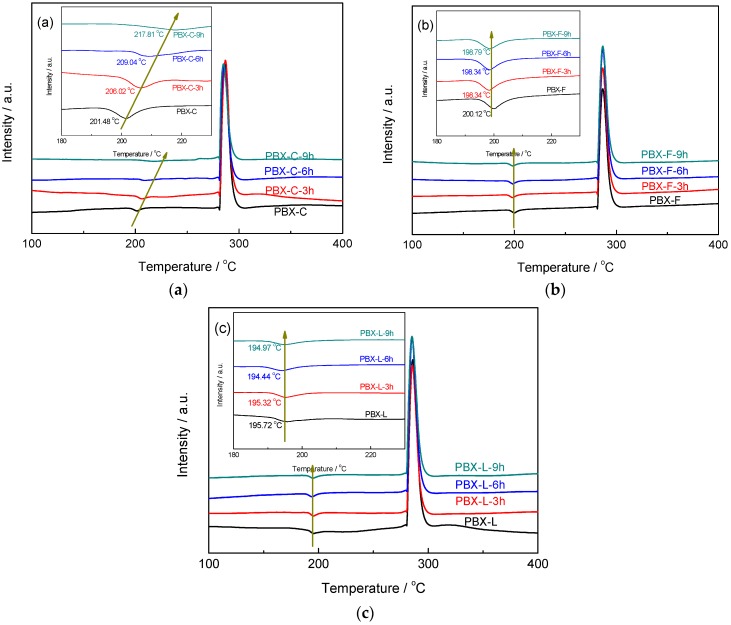
DSC curves of PDA modified PBXs: (**a**) PDA modified PBX-C, (**b**) PDA modified PBX-F, (**c**) PDA modified PBX-L.

Figure 6 and Table S5 in ESI show the DSC results of PDA modified HMX-based PBXs. The DSC curve of PBX-C displayed an endothermic peak at 201.5 °C and an exothermic peak at 286.4 °C, corresponding to the β-δ phase transition and the thermal decomposition of HMX, respectively. The thermal decomposition temperature of HMX for the PDA modified PBXs was almost the same as that for the original PBX material. Notably, the polymorphic phase transition peaks shifted increasingly to 206.0, 209.0, and 217.8 °C for the PDA modified PBX-C samples with PDA coating for 3, 6, and 9 h.

The phase transition behavior of core-shell structured HMX@PDA microcapsules without polymer binder were also studied by DSC analysis to further reveal the phase transition mechanism of PDA modified PBXs. Corresponding results and peak data were demonstrated in Figure S6 and Table S6 in ESI. Compared with the naked C-HMX, the phase transition peak of PBX-C with fluoropolymer as polymer binder showed a slight shift to lower temperature, indicating the unconspicuous role of fluoropolymer towards the phase transition. The phase transition temperature increased with increasing PDA content for core-shell HMX@PDA samples, revealing the enhanced polymorph stability of C-HMX explosives by core-shell coating with PDA. C-HMX@PDA-9h sample exhibited an appreciable increment of temperature peaks by more than 30 °C. Some interesting phenomena were found for PDA modified RS-HMX-based PBXs. Even when the phase transition temperature of HMX for RS-HMX@PDA microcapsules was shifted to ~220 °C, the phase transition temperatures of HMX in PDA modified RS-HMX-based PBXs showed negligible retardation compared with the original RS-HMX-based PBXs.

The difference of coverage degree between the C-HMX@PDA and RS-HMX@PDA composites (Figure 2) could be used to better understand the relationship between the coating structure and thermal phase transition properties of HMX@PDA composites. According to a lattice Monte Carlo simulation, it was found that the morphology of the filler surface was one of the primary factors which influenced the interactions at the interface of polymer matrix-filler particle [41]. PBX was a typical particle-filled polymer-based composite with a high loading of solid explosive crystals. Consequently, the geometry of the energetic crystal surface played a crucial role in determining the interfacial interactions. Besides, compared with the smooth surface of RS-HMX, the rough surface of C-HMX formed an “interlocking block” [40] with PDA to enhance the interfacial interaction.

To further study the relationship between the structure evolvement during the water suspension process and the phase transition of HMX, the effects of solvent, temperature, and stirring on coverage degree of HMX@PDA composites were investigated. As shown in Figure S7 in ESI, after immersion in the ethyl acetate/butyl acetate solvent or water for 1 h or dried in a vacuum oven at 70 °C for 1 h, no distinct difference on the morphology of HMX@PDA composites could be observed, indicating slight influences of solvent, water, and temperature on as-obtained core-shell structure. However, after stirring in the water for 1 h, the surface of HMX@PDA composites cracked. Compared with C-HMX@PDA-9h, stirring could lead to void in the surface of RS-HMX@PDA-9h tending towards both a larger size and higher void concentration. The slight destruction of core-shell structure for C-HMX@PDA-9h after granulation caused the phase transition temperatures of HMX in PBXs to decrease, compared with that of the C-HMX@PDA microcapsules. The high destruction of the core-shell structure during granulation was the main course of no change in the phase transition temperatures of HMX in PDA modified RS-HMX-based PBXs, compared with raw HMX.

### 3.6. Sensitivity Study of PBXs Based on HMX@Polydopamine

The results of impact and friction sensitivity studies of the PDA modified PBXs are summarized in Table 1. The impact sensitivity of PDA modified PBXs remained consistent with raw PBXs (100%), which can be attributed to the following reasons. Firstly, crystal defects were usually responsible for the impact sensitivity, which was enhanced by the growth of hot-spots that originated from crystal defects during impact and adiabatic compression [42]. PDA coating did not change the amounts of defects. Generally, soft coating was helpful to insensitive design by absorbing the impact energy to reduce hot-spots formation. PDA coating was not soft enough to make a difference. Secondly, heat absorbing materials such as wax can alleviate the hot-spots formation during impact, but PDA was not heat absorbing material. Based on these reasons, PDA coating had no effect on hot-spots formation and impact sensitivity.

As shown in Table 1, the friction sensitivity of PDA modified PBXs varied with the crystal quality and particle size of the HMX crystal, depending on the interfaces interaction intension. The introduction of PDA coating had led to more insensitive composites than the original PBX-C. The friction sensitivity of PBX-C-9h samples decreased to 40%, compared with that of the original PBX-C (84%). The same level of sensitivity had been measured for PBX-F samples with and without PDA coating (about 30%). Similar results have been found for the PBX-L used in this study, with a friction sensitivity of 100%. In the friction sensitivity test, the ignition mechanism was a viscous heating of explosive material subjected to extreme velocity gradients as the explosive was violently deformed between the rigid surfaces [43]. This viscous heating mechanism depended on the explosive/polymers interfaces interaction intension. A larger surface roughness of C-HMX benefited a stronger PDA coating; as a result, their interfacial interaction intension was higher than that between PDA coating and RS-HMX, with a smaller surface roughness. The SEM images showed that the PDA coating was damaged in the RS-HMX@PDA coating system during the granulation process, while no similar result was observed in the C-HMX@PDA coating system. One can conclude that PDA benefited the C-HMX/fluoropolymer system as a desensitizer, and not for other systems. A similar example was reported by Bazaki [44]. The catalysts they added in ammonium perchlorate (AP) were responsible for the friction sensitivity by accelerating the AP decomposition, but not responsible for the fall hammer sensitivity.

## 4. Conclusions

In conclusion, PDA coated HMX crystals were synthesized via a facile in situ polymerization of dopamine on the surface of HMX. A compact and uniform PDA coating shell was confirmed by in-depth observation of SEM, XPS, XRD, FT-IR spectra, and Raman spectrum. The core-shell structured HMX@PDA microcapsules were applied to construct PBX with the addition of fluoropolymer. The interface adherence between the HMX crystals and fluoropolymer binder was strengthened with the incorporation of PDA due to the formation of hydrogen bonds and other supramolecular interactions, including π–π stacking and charge transfer. The mechanical properties of as-fabricated HMX@PDA based PBXs showed improved storage modulus, the mechanical strength and toughness, as well as creep resistance. Additionally, the β-δ phase transition temperature of the explosives could be visibly increased by 16.3 °C, attributing to the fact that the rigid PDA shell acted as an armature to protect the HMX crystal. The friction sensitivity could be reduced from 84% to 40% for C-HMX based PBXs with PDA coating. Taking into consideration these results, the preparation of explosive composites with core-shell structure by bio-inspired PDA material provided an effective route for simultaneously boosting mechanical enhancement, thermal stability improvement, and sensitivity reduction of high explosives.

## Figures and Tables

**Table 1:** Impact and friction sensitivities for conventional HMX/fluoropolymer (PBX-C), PDA modified conventional HMX/fluoropolymer with in situ polymerization of dopamine for 9h (PBX-C-9h), fine and high quality HMX/fluoropolymer (PBX-F) and its coating system (PBX-F-9h), large and high quality HMX/fluoropolymer (PBX-L) and its coating system (PBX-L-9h).

Sample	Impact Sensitivity [%]	Friction Sensitivity [%]
PBX-C	96	84
PBX-C-9h	100	40
PBX-F	100	32
PBX-F-9h	100	28
PBX-L	100	100
PBX-L-9h	92	100

## Supplementary Materials

The following are available online at https://www.mdpi.com/2073-4360/11/3/568/s1, Figure S1: OMMRI micrographs of the structure morphologies for different HMX specimens: (a) C-HMX; (b) FRS-HMX; (c) LRS-HMX, Figure S2: Photos of HMX crystals and HMX@PDA composites, Figure S3: The influence of electron beam on the evolution of surface morphology for C-HMX@PDA-9h composites, Figure S4: Characterization of the uncoated HMX crystals and HMX@PDA microparticles: (a) Raman spectra, (b) XRD patterns, (c) FT-IR spectra, and (d) curves of uniaxial stress vs compressive rate, Figure S5: Storage modulus (a) and tanδ (b) as a function of temperature for PDA modified PBXs, Figure S6: DSC curves of HMX@PDA microcapsules, Figure S7: The evolvement in the surface morphology of HMX@PDA microcapsules subjected to solvent, temperature, stirring and water. The solvent was the mixture of ethyl acetate and butyl acetate, and the temperature used is 70 °C, Table S1: The parameters for three types of HMX and HMX@PDA composites, Table S2: Surface element composition of FRS-HMX, PDA, and FRS-HMX @PDA composites as determined by XPS, Table S3: XPS atomic concentration of functional groups in C, O, N atoms of FRS-HMX, PDA, and FRS-HMX@PDA composites, Table S4: Detonation and mechanical characteristics of the PDA modified PBXs, Table S5: Thermal analysis data of the PDA modified PBXs, Table S6: Thermal analysis data of HMX crystals and HMX@PDA microparticles.
