# Supplementary material for: Core-Shell Structured HMX@Polydopamine Energetic Microspheres: Synergistically Enhanced Mechanical, Thermal, and Safety Performances
Source: Polymers (Basel). 2019 Mar 26;11(3):568. doi: 10.3390/polym11030568 (PMC6473810; doi:10.3390/polym11030568)
Supplement: Supplementary file 1 [file polymers-11-00568-s001.pdf]

Electronic Supplementary Information

Core-shell structured HMX@polydopamine energetic microspheres: synergistically enhancing thermal, mechanical and safety performances

Congmei Lin[a,b], Feiyan Gong[a*], Zhijian Yang[a**], Yubin Li[a], Chengcheng Zeng[a], Jiang Li[b], Shaoyun Guo[b]

[a]Institute of Chemical Material, China Academy of Engineering Physics, Mianyang 621900, China

[b]The State Key Laboratory of Polymer Materials Engineering, Polymer Research Institute of Sichuan University, Chengdu 610065, China

E-mail: freya@caep.cn (F. Gong), zhijianyang@caep.cn (Z. Yang).

1 Measurements

1.1 Characterization

Morphological characterization of raw HMX crystals was carried out with an optical microscope with matching refractive index (OMMRI) to qualitatively detect the morphology and defects in HMX crystals. The coating content of PDA shell in the

* Corresponding author.

** Corresponding author.

E-mail addresses: freya@caep.cn (F. Gong), zhijianyang@caep.cn (Z. Yang).

core-shell microcapsules was quantitatively analyzed by an Aqilent 1260 high performance liquid chromatography (HPLC). Morphological study on HMX crystals and HMX@PDA composites was carried out with a scanning electron microscopy (SEM; JSM-6390LV, Zeiss) at an operating voltage of 5 kV. The change of electron binding energy of elements on the HMX surface was detected by X-ray photoelectron energy spectrum (XPS; VG 250, ThermoFisher spectrometer) with monochromatic Mg-Kα X-rays. The particle size distribution of HMX crystals before and after PDA coating was measured by a laser particle size analyzer (LPSA; LS 230, BeckmanCoulter) with a main laser source at a wavelength of 780 nm. Fourier transform infrared (FT-IR) and Raman measurements were performed with an IR spectrometer (Nicolet 6700, ThermoFisher) and FT-Raman spectrometer (Invia-Reflex, Renishaw). X-ray diffraction (XRD) patterns were recorded with a Bruker D8 Advance/LynxEye detector from 5° to 50° with a scan rate of 0.01°/0.2s at 40 kV and 40 mA. The compressive stiffness test (CST) was applied to evaluate the coherence strength of various HMX@PDA composites.

1.2 Mechanical testing

The compressive and Brazilian tests of the specimens were performed using an Instron 5582 machine (Canton, MA, USA) at a room temperature. The crosshead speed was set at 10 mm/min. At least three specimens of each PBX were tested, and the average values were reported. A confined, quasi-static uniaxial compression method, named as the compressive stiffness test (CST), was promoted to evaluate the quality of HMX@PDA particles. Dynamic mechanical analyzer (DMA 242C, Netzsch,

Germany) was used to conduct the DMA tests and creep tests in a three-point bending mode. The specimen dimensions were 30 mm × 10 mm × 1-2 mm (length × width × thickness).

1.3 Thermal analysis

The thermal analysis was determined with a Mettler Toledo instrument by thermogravimetric-differential scanning calorimetry (TG-DSC). The data were collected from 40 to 600 °C at a heating rate of 10 °C/min in a nitrogen (40 mL/min) atmosphere.

1.4 Sensitivity tests

According to GJB-772A-97 standard method 601.2 and 602.1 [1], the impact and friction sensitivity tests of PBXs composites were conducted with a WL-1 type impact sensitivity instrument and a WM-1 type friction sensitivity instrument. The impact sensitivity test conditions were: drop weight, 10 kg; drop height, 25 cm; sample mass, 50 mg. The friction sensitivity test conditions were: relative pressure, 3.92 MPa; sample mass: 30 mg, pendulum weight: 1.5 kg; pendulum angle: 90°. The impact and friction sensitivity of each test sample was expressed by explosion probability (P).

2. Supplemented Tables and Figures

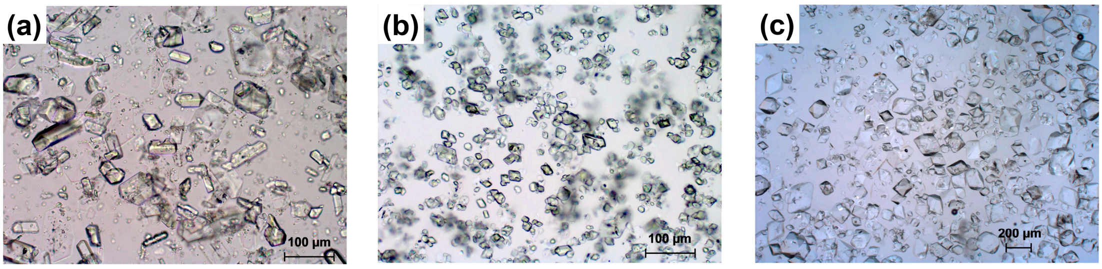

Fig. S1. OMMRI micrographs of the structure morphologies for different HMX specimens: (a) C-HMX; (b) FRS-HMX; (c) LRS-HMX.

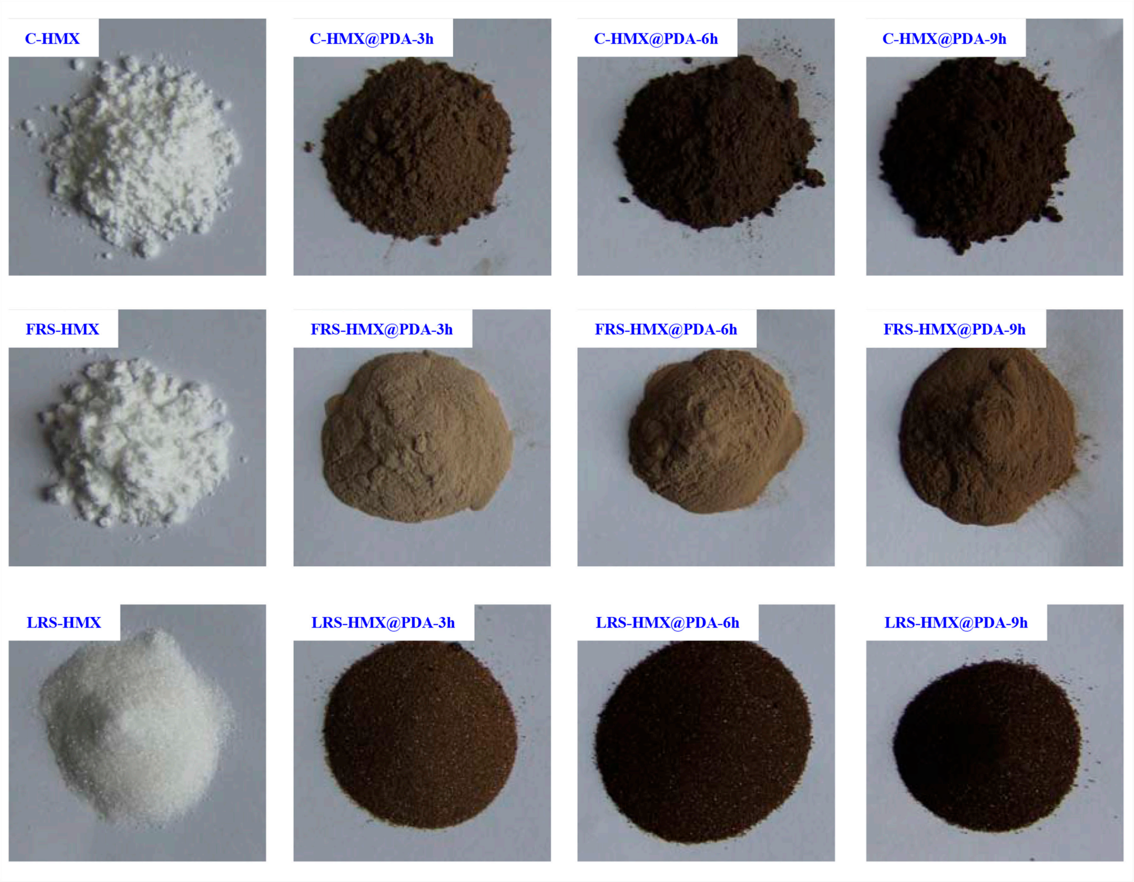

Fig. S2. Photos of HMX crystals and HMX@PDA composites**.**

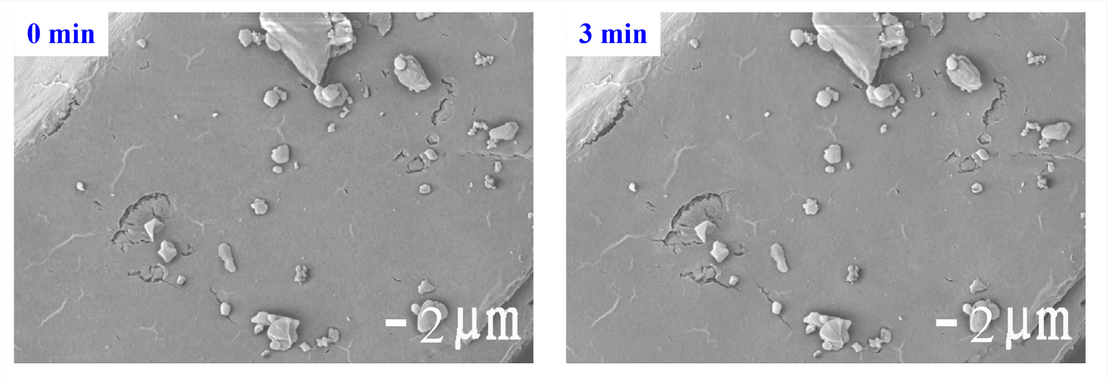

Fig. S3. The influence of electron beam on the evolution of surface morphology for C-HMX@PDA-9h composites.

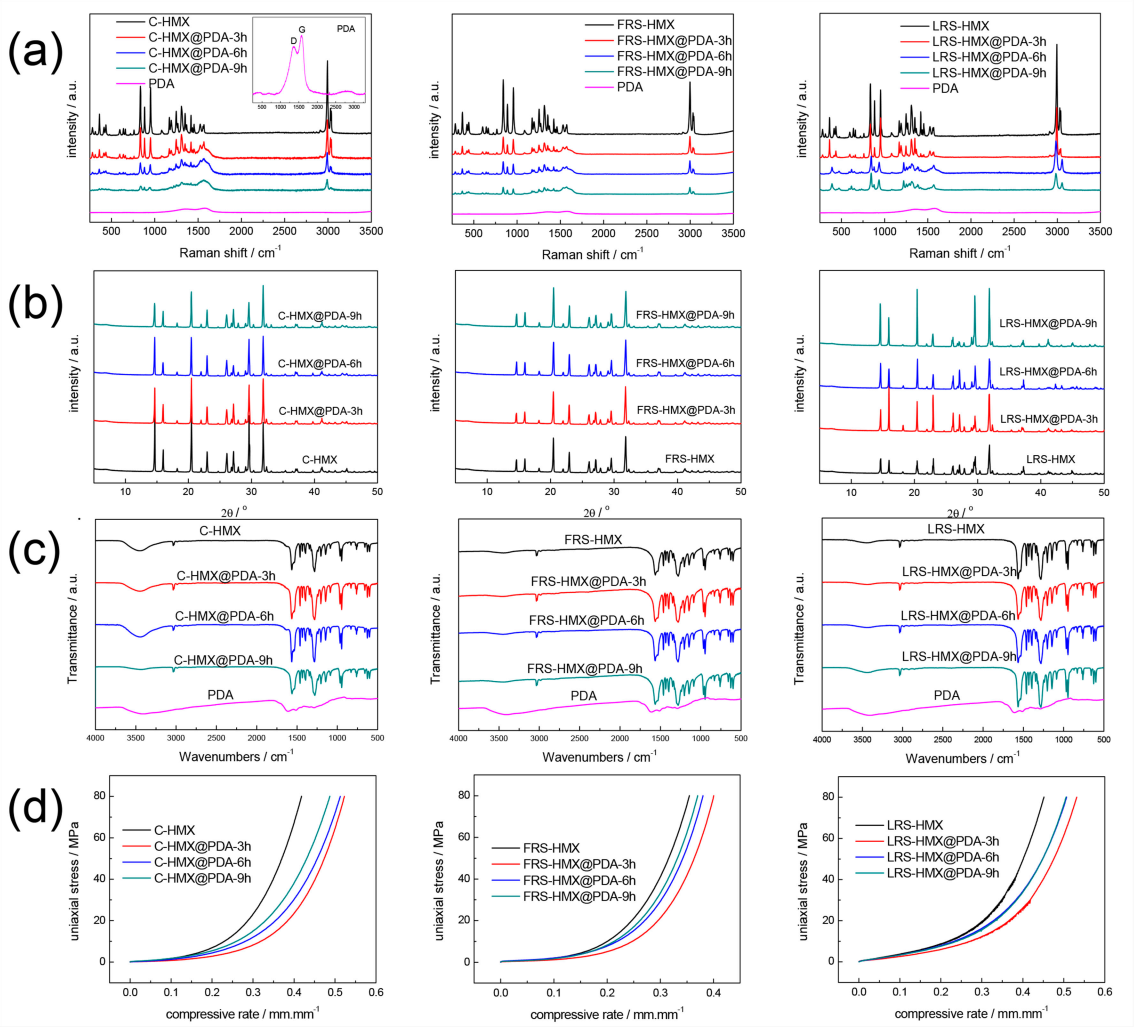

Fig. S4. Characterization of the uncoated HMX crystals and HMX@PDA microparticles: (a) Raman spectra, (b) XRD patterns, (c) FT-IR spectra, and (d) curves of uniaxial stress vs compressive rate.

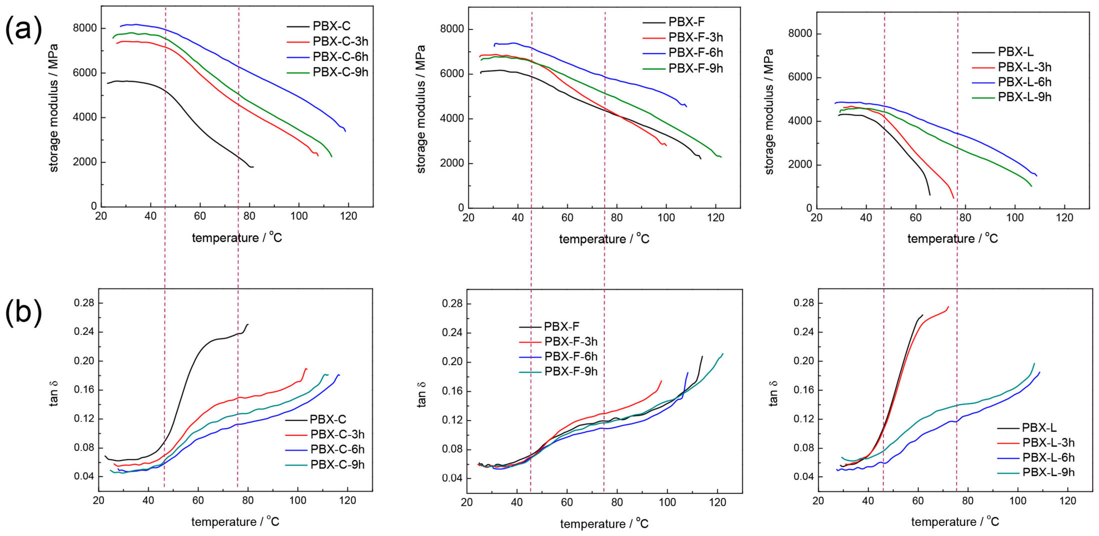

Fig. S5. Storage modulus (a) and tanδ (b) as a function of temperature for PDA modified PBXs.

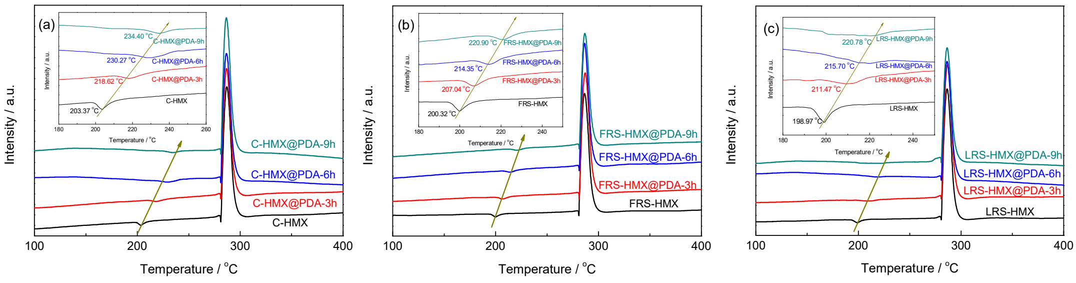

Fig. S6. DSC curves of HMX@PDA microcapsules.

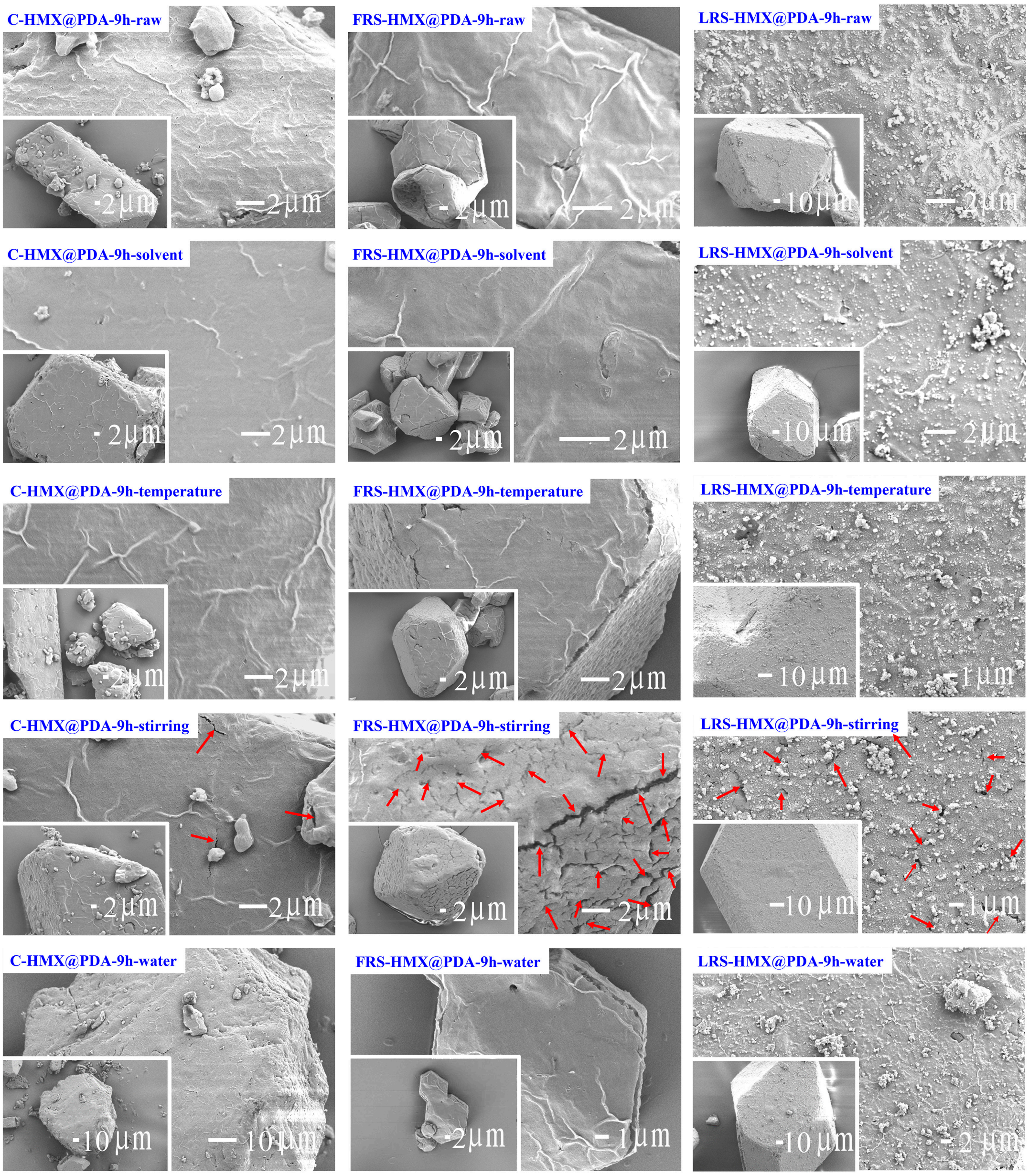

Fig. S7. The evolvement in the surface morphology of HMX@PDA microcapsules subjected to solvent, temperature, stirring and water. The solvent was the mixture of ethyl acetate and butyl acetate, and the temperature used is 70 °C.

Table S1 The parameters for three types of HMX and HMX@PDA composites.

Sample	PDA content [%]	Average particle size [μm]	ISM[a] [MPa]
C-HMX	0	47.0±1.96	99.30
C-HMX@PDA-3h	0.75	49.2±2.53	78.44
C-HMX@PDA-6h	0.95	49.4±1.21	83.90
C-HMX@PDA-9h	1.43	51.4±1.78	91.46
FRS-HMX	0	44.8±1.48	123.14
FRS-HMX@PDA-3h	0.11	33.1±1.23	101.73
FRS-HMX@PDA-6h	0.20	32.8±0.47	103.66
FRS-HMX@PDA-9h	0.50	30.4±2.48	118.28
LRS-HMX	0	149.1±4.75	104.10
LRS-HMX@PDA-3h	0.30	141.2±4.65	76.24
LRS-HMX@PDA-6h	0.36	138.4±2.79	88.85
LRS-HMX@PDA-9h	0.49	145.0±6.96	92.59

[a] ISM represents the initial secant modulus to evaluate the coherence strength of energetic crystalline granules.

Table S2

Surface element composition of FRS-HMX, PDA, and FRS-HMX @PDA composites as determined by XPS.

Samples	C1s (%)	O1s (%)	N1s (%)	N/C
FRS-HMX	32.57	32.09	35.34	1.085
PDA	70.85	21.33	7.82	0.110
FRS-HMX@PDA-3h	50.06	25.67	24.27	0.485
FRS-HMX@PDA-6h	55.58	25.46	18.96	0.341
FRS-HMX@PDA-9h	60.75	21.84	17.41	0.287

Table S3

XPS atomic concentration of functional groups in C, O, N atoms of FRS-HMX, PDA, and FRS-HMX@PDA composites.

Atom	Peak position (eV)	Assignment	FRS-HMX	PDA	FRS-HMX @PDA-3h	FRS-HMX @PDA-6h	FRS-HMX @PDA-9h
C 1s	284.6	C-H, C-C,	12.58	32.59	22.70	21.06	24.32
	285.6-285.9	C-O, C-N	--	27.40	10.16	19.20	23.76
	287.8-288.3	C=O, N-C-N	19.99	9.63	14.94	15.31	11.62
	291.1-291.3	π-π*shake up	--	1.23	2.26	0.02	1.05
N 1s	398.3-399.1	C-N=C	--	5.77	0.68	0.35	0.66
	399.9-400.0	C-NH-C	--	12.02	2.23	3.89	4.23
	401.4-402.1	C-N, C-NH_2	16.76	3.54	12.03	11.46	9.63
	407.1-407.8	-NO_2	15.33	--	10.72	9.76	7.32
O 1s	531.1-532.6	C=O	--	2.26	9.18	8.11	2.23
	532.9-533.7	-NO_2, C-OH	35.34	5.56	15.09	10.85	15.18

Table S4 Detonation and mechanical characteristics of the PDA modified PBXs

Sample	Calculated theoretical detonation velocity (m/s)	Compressive strength (MPa)	Compressive modulus (GPa)	Compressive elongation at break (%)	W_c ($\times 10^4$ J.m^{-3})	Tensile strength (MPa)	Tensile modulus (GPa)	Tensile elongation at break (%)	W_t ($\times 10^4$ J.m^{-3})
PBX-C	8972.4	47.51±0.06	3.23±0.08	2.32±0.01	64.96±0.788	5.56±0.12	11.76±0.17	0.080±0.003	0.279±0.003
PBX-C-3h	8957.4	55.33±0.31	3.89±0.09	2.15±0.01	68.06±0.156	5.88±0.25	9.81±0.43	0.097±0.002	0.368±0.006
PBX-C-6h	8953.4	56.11±0.30	3.72±0.30	2.14±0.13	70.59±0.275	6.29±0.24	11.52±0.86	0.096±0.011	0.391±0.015
PBX-C-9h	8943.7	54.44±0.01	3.61±0.18	2.10±0.13	68.90±0.764	6.05±0.15	11.09±0.39	0.095±0.002	0.367±0.013
PBX-F	8972.4	56.43±0.96	3.21±0.09	1.96±0.01	58.20±0.487	5.87±0.29	8.11±0.38	0.096±0.009	0.333±0.008
PBX-F-3h	8970.2	59.19±0.43	3.38±0.09	2.22±0.02	64.37±0.136	6.08±0.03	8.75±0.53	0.104±0.003	0.387±0.012
PBX-F-6h	8968.4	60.96±0.03	3.48±0.04	2.10±0.09	66.09±0.151	6.20±0.04	9.94±0.10	0.103±0.009	0.391±0.005
PBX-F-9h	8962.4	61.69±0.72	3.36±0.11	2.10±0.08	65.04±0.224	6.08±0.05	9.20±0.16	0.097±0.017	0.358±0.023
PBX-L	8972.4	29.00±0.14	2.52±0.09	1.87±0.09	32.30±0.881	3.02±0.09	9.90±0.80	0.048±0.004	0.093±0.011
PBX-L-3h	8966.4	31.15±0.56	2.25±0.01	2.07±0.01	36.80±0.175	3.34±0.14	9.33±0.93	0.050±0.002	0.106±0.005
PBX-L-6h	8965.2	34.28±0.09	2.12±0.02	2.30±0.02	39.41±0.550	3.50±0.18	9.94±0.36	0.056±0.007	0.129±0.016
PBX-L-9h	8962.6	31.88±0.21	2.37±0.05	2.06±0.05	37.91±0.153	3.20±0.14	9.96±0.86	0.050±0.008	0.100±0.017

Table S5 Thermal analysis data of the PDA modified PBXs

Sample	Endothermic peak				Exothermic peak			
	T_o / °C	T_p / °C	T_e / °C	△H /J.g^{-1}	T_o / °C	T_p / °C	T_e / °C	△H /J.g^{-1}
PBX-C	195.41	201.48	208.49	-38.68	281.53	286.43	293.93	1732.16
PBX-C-3h	200.18	206.02	213.40	-38.24	281.62	286.81	293.17	1582.67
PBX-C-6h	203.32	209.04	226.07	-33.53	281.07	285.70	292.39	1330.26
PBX-C-9h	202.96	217.81	230.48	-38.71	281.09	285.05	292.12	1200.52
PBX-F	195.13	200.12	206.44	-43.49	281.64	286.64	294.08	1669.54
PBX-F-3h	193.46	198.34	204.62	-40.20	281.62	286.60	294.25	1723.59
PBX-F-6h	193.43	198.34	204.66	-47.97	281.88	286.66	294.21	1845.53
PBX-F-9h	193.56	198.79	205.38	-33.23	281.75	286.65	294.00	1650.92
PBX-L	190.54	195.72	199.1	-47.67	280.73	285.93	293.72	1784.63
PBX-L-3h	190.93	195.32	202.10	-34.15	280.71	285.36	293.56	1725.77
PBX-L-6h	188.76	194.44	201.22	-39.01	280.80	285.39	292.95	1787.68
PBX-L-9h	189.68	194.97	202.90	-35.96	280.29	285.16	293.54	1656.74

Table S6 Thermal analysis data of HMX crystals and HMX@PDA microparticles.

Sample	PDA Content %	Endothermic peak				Exothermic peak			
		T_o / °C	T_p / °C	T_e / °C	△H /J.g^{-1}	T_o / °C	T_p / °C	T_e / °C	△H /J.g^{-1}
C-HMX	0	199.17	203.37	209.96	-41.68	281.40	286.99	294.29	1751.63
C-HMX@PDA-3h	0.75	210.38	218.62	229.77	-48.57	281.35	286.78	294.27	1815.94
C-HMX@PDA-6h	0.95	218.70	230.47	241.27	-50.49	281.31	286.52	294.11	1773.53
C-HMX@PDA-9h	1.43	224.41	234.23	243.52	-48.35	281.45	286.51	293.96	1890.32
FRS-HMX	0	195.42	200.32	206.67	-47.29	281.44	286.97	294.54	1845.44
FRS-HMX@PDA-3h	0.11	200.48	207.04	215.08	-51.03	281.49	287.01	294.048	1844.73
FRS-HMX@PDA-6h	0.20	206.90	214.35	222.00	-44.72	281.56	286.27	294.08	1781.64
FRS-HMX@PDA-9h	0.50	213.99	220.90	229.62	-32.70	281.58	286.72	294.35	1780.46
LRS-HMX	0	193.26	198.97	204.23	-43.82	280.79	286.41	296.04	1774.53
LRS-HMX@PDA-3h	0.30	195.79	211.47	222.53	-53.07	280.98	286.61	294.30	1849.00
LRS-HMX@PDA-6h	0.36	211.97	215.70	227.28	-41.54	281.31	286.52	294.11	1773.53
LRS-HMX@PDA-9h	0.49	204.70	219.05	231.49	-42.55	281.45	286.51	293.96	1890.72

References

[1] National Military Standard of China, Experimental Methods of Sensitivity and Safety, GJB/772A-97, 1997 (in Chinese).
